# Supplementary material for: Adsorption Characteristics of Ag Nanoparticles on Cellulose Nanofibrils with Different Chemical Compositions
Source: Polymers (Basel). 2020 Jan 8;12(1):164. doi: 10.3390/polym12010164 (PMC7023221; doi:10.3390/polym12010164)
Supplement: Supplementary file 1 [file polymers-12-00164-s001.pdf]

Supporting information

Adsorption Characteristics of Ag Nanoparticles on Cellulose Nanofibrils with Different Chemical Compositions

Gu-Joong Kwon [1], Song-Yi Han [2], Chan-Woo Park [2], Ji-Soo Park [2], En-Ah Lee [2], Nam-Hun Kim [2], Madhusudhan Alle [3], Rajkumar Bandi [3] and Seung-Hwan Lee [2,*]

[1] Kangwon Institute of Inclusion Technology, Kangwon National University, Chuncheon-Si, Gangwon-do, 24341, Republic of Korea; gjkwon@kangwon.ac.kr(G.-J.K.)

[2] Department of Forest Biomaterials Engineering, College of Forest and Environmental Sciences, Kangwon National University, Chuncheon-si, Gangwon-do, 24341, Republic of Korea; songlee618@kangwon.ac.kr(S.-Y.H.);chanwoo8973@kangwon.ac.kr(C.-W.P.);pojs04@kangwon.ac.kr(J.-S.P.);laa3158@kangwon.ac.kr(E.-A.L.);nhkim@kangwon.ac.kr(N.-H.K); ishyhk@ kangwon.ac.kr(S.-H.L.)

[3] Institute of Forest Science, Kangwon National University, Chuncheon-si, Gangwon-do, 24341, Republic of Korea; allemadhusudhan@gmail.com(M.A.); rajkumar.pgcb@gmail.com(R.B.)

* Correspondence: lshyhk@kangwon.ac.kr(S.-H.L.); Tel.:+82-33-250-8323

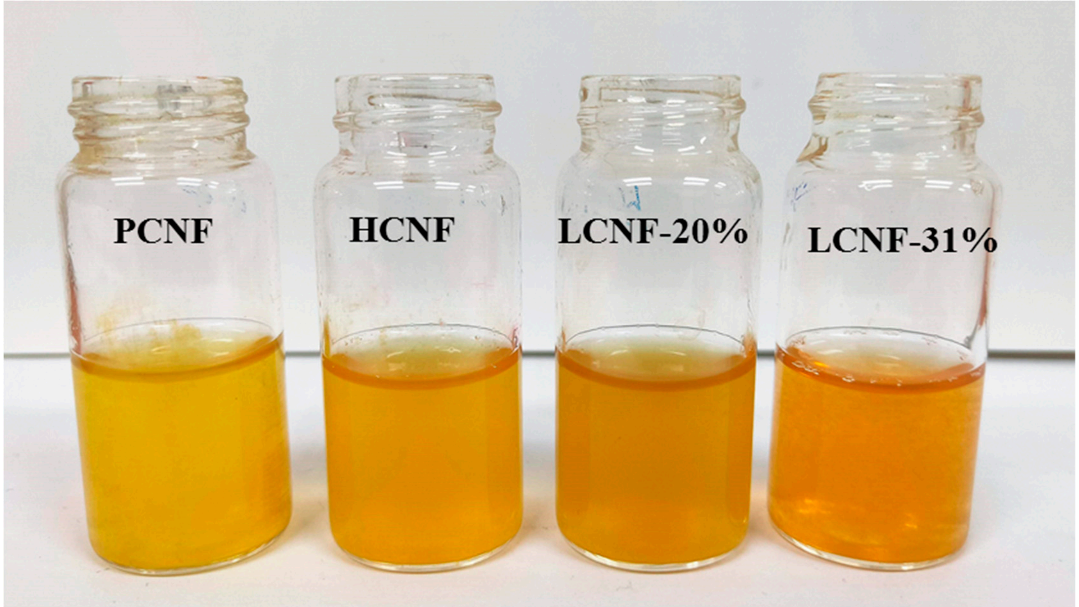

Figure S1. Digital photograph showing the aqueous dispersions of AgNPs adsorbed on different CNFs (0.1 wt%).
